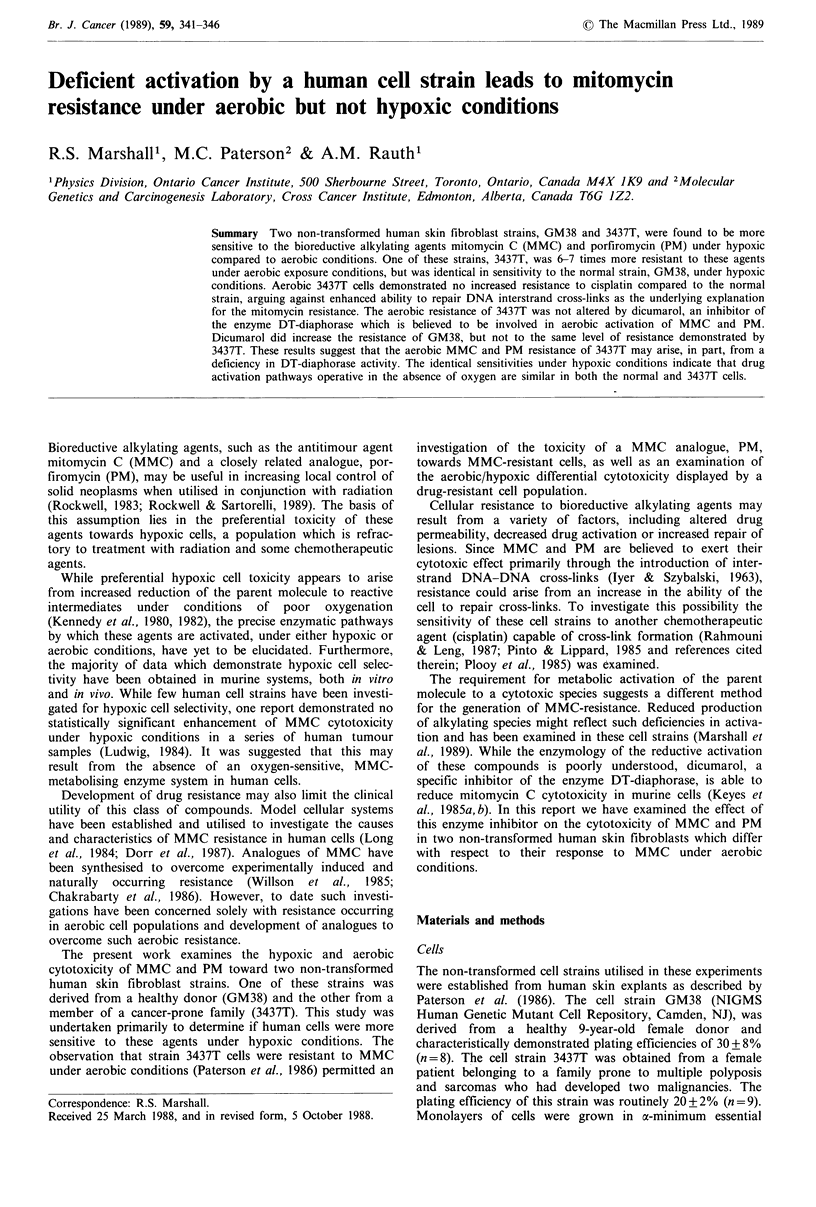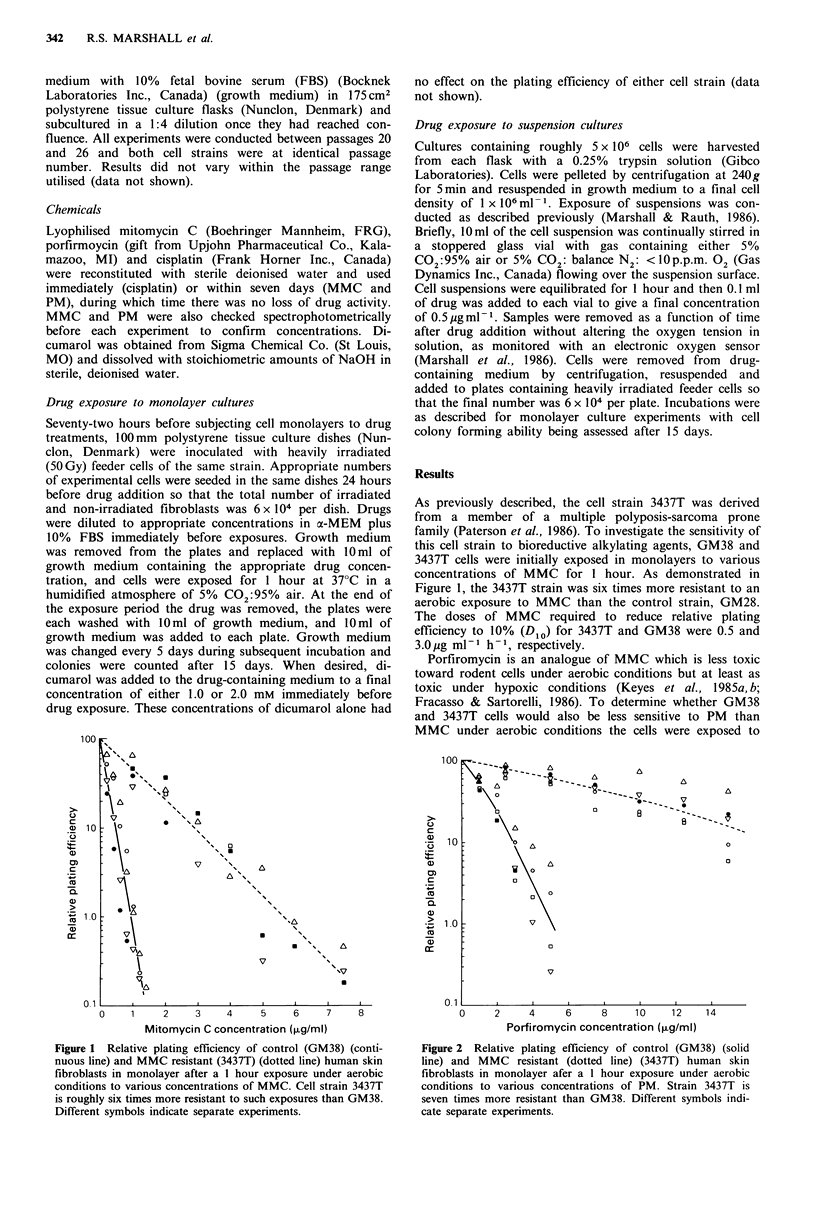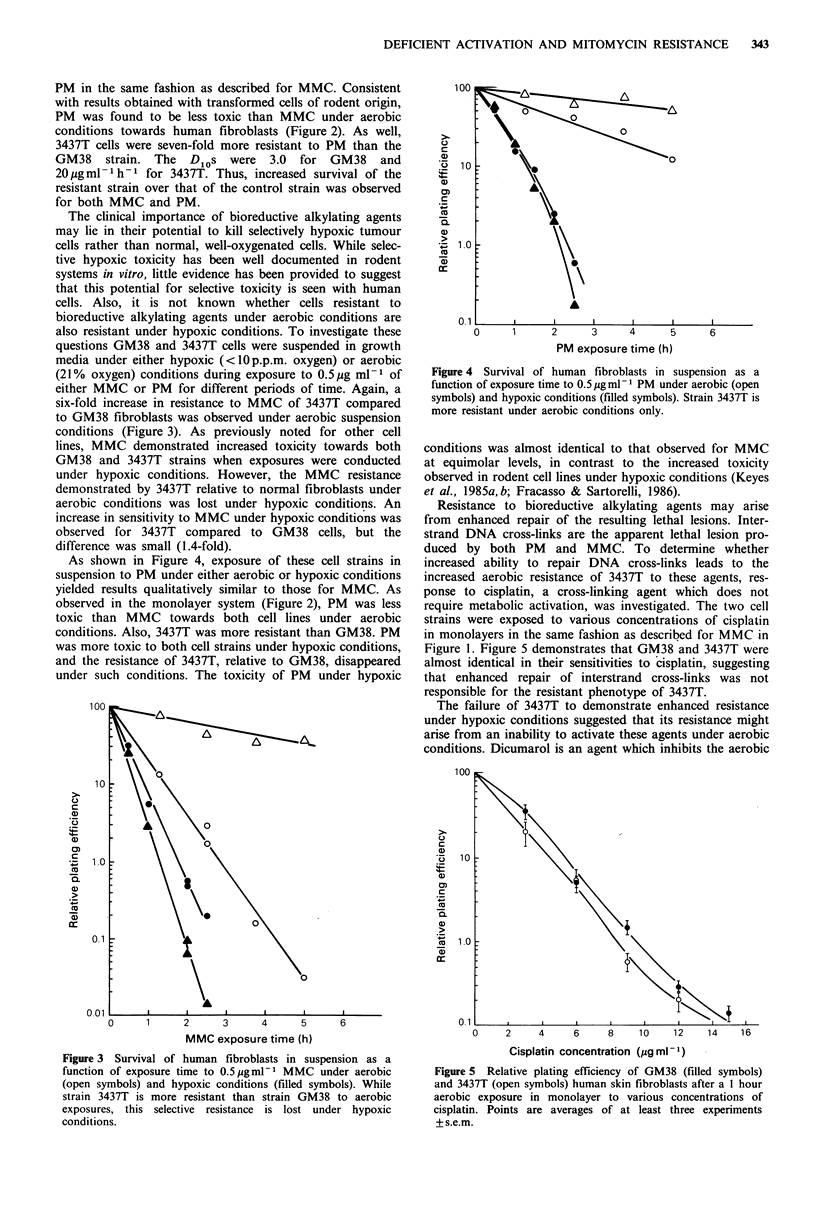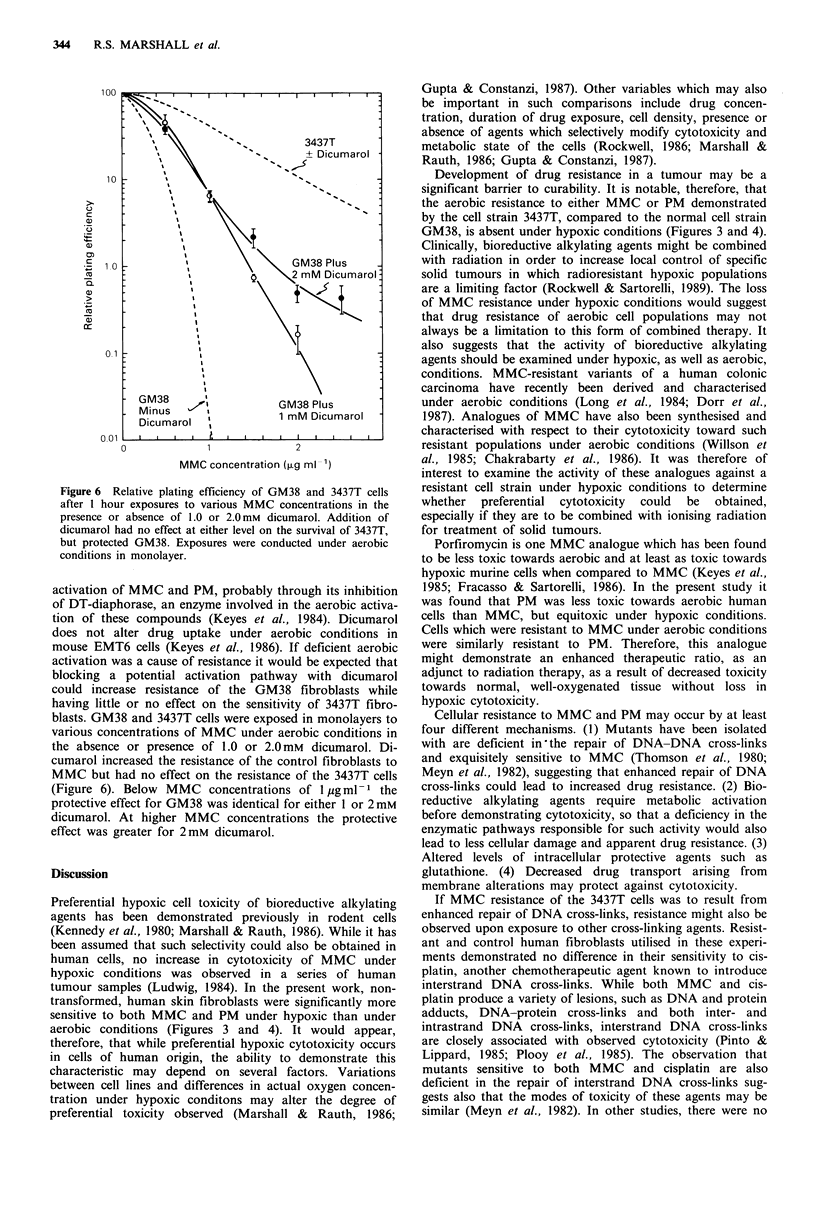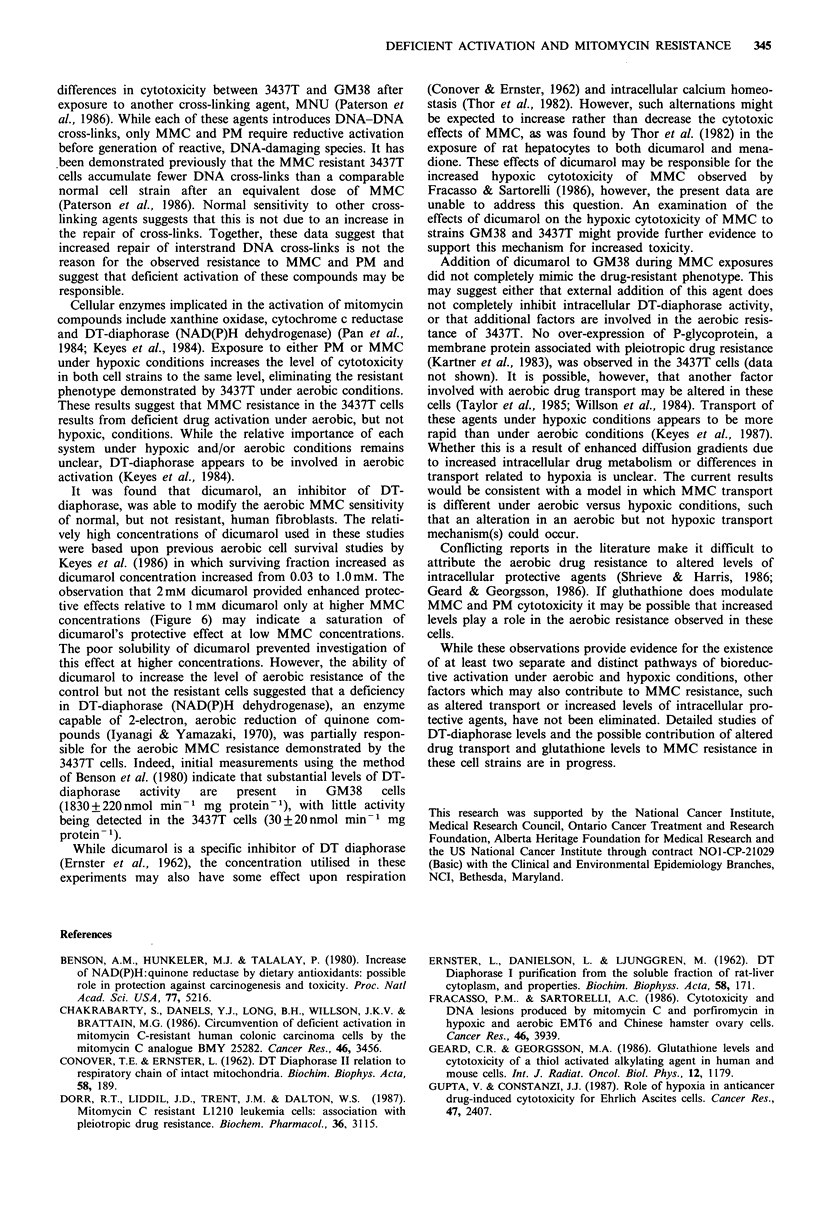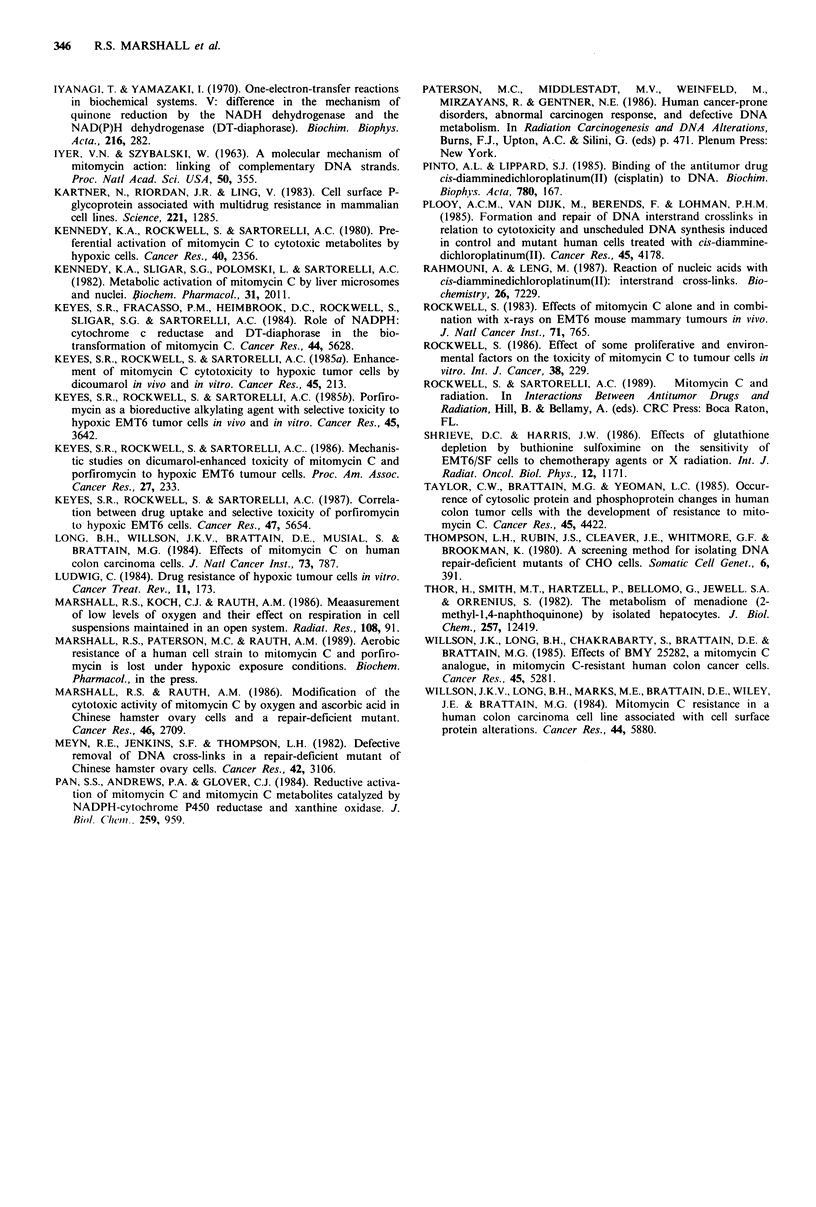# Deficient activation by a human cell strain leads to mitomycin resistance under aerobic but not hypoxic conditions.

**DOI:** 10.1038/bjc.1989.67

**Published:** 1989-03

**Authors:** R. S. Marshall, M. C. Paterson, A. M. Rauth

**Affiliations:** Physics Division, Ontario Cancer Institute, Toronto, Canada.

## Abstract

Two non-transformed human skin fibroblast strains, GM38 and 3437T, were found to be more sensitive to the bioreductive alkylating agents mitomycin C (MMC) and porfiromycin (PM) under hypoxic compared to aerobic conditions. One of these strains, 3437T, was 6-7 times more resistant to these agents under aerobic exposure conditions, but was identical in sensitivity to the normal strain, GM38, under hypoxic conditions. Aerobic 3437T cells demonstrated no increased resistance to cisplatin compared to the normal strain, arguing against enhanced ability to repair DNA interstrand cross-links as the underlying explanation for the mitomycin resistance. The aerobic resistance of 3437T was not altered by dicumarol, an inhibitor of the enzyme DT-diaphorase which is believed to be involved in aerobic activation of MMC and PM. Dicumarol did increase the resistance of GM38, but not to the same level of resistance demonstrated by 3437T. These results suggest that the aerobic MMC and PM resistance of 3437T may arise, in part, from a deficiency in DT-diaphorase activity. The identical sensitivities under hypoxic conditions indicate that drug activation pathways operative in the absence of oxygen are similar in both the normal and 3437T cells.


Br. J. Cancer (1989), 59, 341–346 © The Macmillan Press Ltd., 1989

### Deficient activation by a human cell strain leads to mitomycin resistance under aerobic but not hypoxic conditions

R.S. Marshall[1], M.C. Paterson[2] & A.M. Rauth[1]

[1]Physics Division, Ontario Cancer Institute, 500 Sherbourne Street, Toronto, Ontario, Canada M4X 1K9 and [2]Molecular Genetics and Carcinogenesis Laboratory, Cross Cancer Institute, Edmonton, Alberta, Canada T6G 1Z2.

**Summary** Two non-transformed human skin fibroblast strains, GM38 and 3437T, were found to be more sensitive to the bioreductive alkylating agents mitomycin C (MMC) and porfiromycin (PM) under hypoxic compared to aerobic conditions. One of these strains, 3437T, was 6–7 times more resistant to these agents under aerobic exposure conditions, but was identical in sensitivity to the normal strain, GM38, under hypoxic conditions. Aerobic 3437T cells demonstrated no increased resistance to cisplatin compared to the normal strain, arguing against enhanced ability to repair DNA interstrand cross-links as the underlying explanation for the mitomycin resistance. The aerobic resistance of 3437T was not altered by dicumarol, an inhibitor of the enzyme DT-diaphorase which is believed to be involved in aerobic activation of MMC and PM. Dicumarol did increase the resistance of GM38, but not to the same level of resistance demonstrated by 3437T. These results suggest that the aerobic MMC and PM resistance of 3437T may arise, in part, from a deficiency in DT-diaphorase activity. The identical sensitivities under hypoxic conditions indicate that drug activation pathways operative in the absence of oxygen are similar in both the normal and 3437T cells.

Bioreductive alkylating agents, such as the antitimour agent mitomycin C (MMC) and a closely related analogue, porfiromycin (PM), may be useful in increasing local control of solid neoplasms when utilised in conjunction with radiation (Rockwell, 1983; Rockwell & Sartorelli, 1989). The basis of this assumption lies in the preferential toxicity of these agents towards hypoxic cells, a population which is refractory to treatment with radiation and some chemotherapeutic agents.

While preferential hypoxic cell toxicity appears to arise from increased reduction of the parent molecule to reactive intermediates under conditions of poor oxygenation (Kennedy *et al.*, 1980, 1982), the precise enzymatic pathways by which these agents are activated, under either hypoxic or aerobic conditions, have yet to be elucidated. Furthermore, the majority of data which demonstrate hypoxic cell selectivity have been obtained in murine systems, both *in vitro* and *in vivo*. While few human cell strains have been investigated for hypoxic cell selectivity, one report demonstrated no statistically significant enhancement of MMC cytotoxicity under hypoxic conditions in a series of human tumour samples (Ludwig, 1984). It was suggested that this may result from the absence of an oxygen-sensitive, MMC-metabolising enzyme system in human cells.

Development of drug resistance may also limit the clinical utility of this class of compounds. Model cellular systems have been established and utilised to investigate the causes and characteristics of MMC resistance in human cells (Long *et al.*, 1984; Dorr *et al.*, 1987). Analogues of MMC have been synthesised to overcome experimentally induced and naturally occurring resistance (Willson *et al.*, 1985; Chakrabarty *et al.*, 1986). However, to date such investigations have been concerned solely with resistance occurring in aerobic cell populations and development of analogues to overcome such aerobic resistance.

The present work examines the hypoxic and aerobic cytotoxicity of MMC and PM toward two non-transformed human skin fibroblast strains. One of these strains was derived from a healthy donor (GM38) and the other from a member of a cancer-prone family (3437T). This study was undertaken primarily to determine if human cells were more sensitive to these agents under hypoxic conditions. The observation that strain 3437T cells were resistant to MMC under aerobic conditions (Paterson *et al.*, 1986) permitted an investigation of the toxicity of a MMC analogue, PM, towards MMC-resistant cells, as well as an examination of the aerobic/hypoxic differential cytotoxicity displayed by a drug-resistant cell population.

Cellular resistance to bioreductive alkylating agents may result from a variety of factors, including altered drug permeability, decreased drug activation or increased repair of lesions. Since MMC and PM are believed to exert their cytotoxic effect primarily through the introduction of interstrand DNA–DNA cross-links (Iyer & Szybalski, 1963), resistance could arise from an increase in the ability of the cell to repair cross-links. To investigate this possibility the sensitivity of these cell strains to another chemotherapeutic agent (cisplatin) capable of cross-link formation (Rahmouni & Leng, 1987; Pinto & Lippard, 1985 and references cited therein; Plooy *et al.*, 1985) was examined.

The requirement for metabolic activation of the parent molecule to a cytotoxic species suggests a different method for the generation of MMC-resistance. Reduced production of alkylating species might reflect such deficiencies in activation and has been examined in these cell strains (Marshall *et al.*, 1989). While the enzymology of the reductive activation of these compounds is poorly understood, dicumarol, a specific inhibitor of the enzyme DT-diaphorase, is able to reduce mitomycin C cytotoxicity in murine cells (Keyes *et al.*, 1985*a,b*). In this report we have examined the effect of this enzyme inhibitor on the cytotoxicity of MMC and PM in two non-transformed human skin fibroblasts which differ with respect to their response to MMC under aerobic conditions.

### Materials and methods

### Cells

The non-transformed cell strains utilised in these experiments were established from human skin explants as described by Paterson *et al.* (1986). The cell strain GM38 (NIGMS Human Genetic Mutant Cell Repository, Camden, NJ), was derived from a healthy 9-year-old female donor and characteristically demonstrated plating efficiencies of $30 \pm 8\%$ ($n = 8$). The cell strain 3437T was obtained from a female patient belonging to a family prone to multiple polyposis and sarcomas who had developed two malignancies. The plating efficiency of this strain was routinely $20 \pm 2\%$ ($n = 9$). Monolayers of cells were grown in α-minimum essential

Correspondence: R.S. Marshall.
Received 25 March 1988, and in revised form, 5 October 1988.

medium with 10% fetal bovine serum (FBS) (Bocknek Laboratories Inc., Canada) (growth medium) in 175 cm$^2$ polystyrene tissue culture flasks (Nunclon, Denmark) and subcultured in a 1:4 dilution once they had reached confluence. All experiments were conducted between passages 20 and 26 and both cell strains were at identical passage number. Results did not vary within the passage range utilised (data not shown).

*Chemicals*

Lyophilised mitomycin C (Boehringer Mannheim, FRG), porfirmoycin (gift from Upjohn Pharmaceutical Co., Kalamazoo, MI) and cisplatin (Frank Horner Inc., Canada) were reconstituted with sterile deionised water and used immediately (cisplatin) or within seven days (MMC and PM), during which time there was no loss of drug activity. MMC and PM were also checked spectrophotometrically before each experiment to confirm concentrations. Dicumarol was obtained from Sigma Chemical Co. (St Louis, MO) and dissolved with stoichiometric amounts of NaOH in sterile, deionised water.

*Drug exposure to monolayer cultures*

Seventy-two hours before subjecting cell monolayers to drug treatments, 100 mm polystyrene tissue culture dishes (Nunclon, Denmark) were inoculated with heavily irradiated (50 Gy) feeder cells of the same strain. Appropriate numbers of experimental cells were seeded in the same dishes 24 hours before drug addition so that the total number of irradiated and non-irradiated fibroblasts was $6 \times 10^4$ per dish. Drugs were diluted to appropriate concentrations in α-MEM plus 10% FBS immediately before exposures. Growth medium was removed from the plates and replaced with 10 ml of growth medium containing the appropriate drug concentration, and cells were exposed for 1 hour at 37°C in a humidified atmosphere of 5% $CO_2$:95% air. At the end of the exposure period the drug was removed, the plates were each washed with 10 ml of growth medium, and 10 ml of growth medium was added to each plate. Growth medium was changed every 5 days during subsequent incubation and colonies were counted after 15 days. When desired, dicumarol was added to the drug-containing medium to a final concentration of either 1.0 or 2.0 mM immediately before drug exposure. These concentrations of dicumarol alone had no effect on the plating efficiency of either cell strain (data not shown).

*Drug exposure to suspension cultures*

Cultures containing roughly $5 \times 10^6$ cells were harvested from each flask with a 0.25% trypsin solution (Gibco Laboratories). Cells were pelleted by centrifugation at 240 *g* for 5 min and resuspended in growth medium to a final cell density of $1 \times 10^6$ ml$^{-1}$. Exposure of suspensions was conducted as described previously (Marshall & Rauth, 1986). Briefly, 10 ml of the cell suspension was continually stirred in a stoppered glass vial with gas containing either 5% $CO_2$:95% air or 5% $CO_2$: balance $N_2$: <10 p.p.m. $O_2$ (Gas Dynamics Inc., Canada) flowing over the suspension surface. Cell suspensions were equilibrated for 1 hour and then 0.1 ml of drug was added to each vial to give a final concentration of 0.5 μg ml$^{-1}$. Samples were removed as a function of time after drug addition without altering the oxygen tension in solution, as monitored with an electronic oxygen sensor (Marshall *et al.*, 1986). Cells were removed from drug-containing medium by centrifugation, resuspended and added to plates containing heavily irradiated feeder cells so that the final number was $6 \times 10^4$ per plate. Incubations were as described for monolayer culture experiments with cell colony forming ability being assessed after 15 days.

### Results

As previously described, the cell strain 3437T was derived from a member of a multiple polyposis-sarcoma prone family (Paterson *et al.*, 1986). To investigate the sensitivity of this cell strain to bioreductive alkylating agents, GM38 and 3437T cells were initially exposed in monolayers to various concentrations of MMC for 1 hour. As demonstrated in Figure 1, the 3437T strain was six times more resistant to an aerobic exposure to MMC than the control strain, GM28. The doses of MMC required to reduce relative plating efficiency to 10% ($D_{10}$) for 3437T and GM38 were 0.5 and 3.0 μg ml$^{-1}$ h$^{-1}$, respectively.

Porfiromycin is an analogue of MMC which is less toxic toward rodent cells under aerobic conditions but at least as toxic under hypoxic conditions (Keyes *et al.*, 1985*a*,*b*; Fracasso & Sartorelli, 1986). To determine whether GM38 and 3437T cells would also be less sensitive to PM than MMC under aerobic conditions the cells were exposed to

**Figure 1** Relative plating efficiency of control (GM38) (continuous line) and MMC resistant (3437T) (dotted line) human skin fibroblasts in monolayer after a 1 hour exposure under aerobic conditions to various concentrations of MMC. Cell strain 3437T is roughly six times more resistant to such exposures than GM38. Different symbols indicate separate experiments.

**Figure 2** Relative plating efficiency of control (GM38) (solid line) and MMC resistant (dotted line) (3437T) human skin fibroblasts in monolayer afer a 1 hour exposure under aerobic conditions to various concentrations of PM. Strain 3437T is seven times more resistant than GM38. Different symbols indicate separate experiments.

PM in the same fashion as described for MMC. Consistent with results obtained with transformed cells of rodent origin, PM was found to be less toxic than MMC under aerobic conditions towards human fibroblasts (Figure 2). As well, 3437T cells were seven-fold more resistant to PM than the GM38 strain. The $D_{10}$s were 3.0 for GM38 and $20 \mu g ml^{-1} h^{-1}$ for 3437T. Thus, increased survival of the resistant strain over that of the control strain was observed for both MMC and PM.

The clinical importance of bioreductive alkylating agents may lie in their potential to kill selectively hypoxic tumour cells rather than normal, well-oxygenated cells. While selective hypoxic toxicity has been well documented in rodent systems *in vitro*, little evidence has been provided to suggest that this potential for selective toxicity is seen with human cells. Also, it is not known whether cells resistant to bioreductive alkylating agents under aerobic conditions are also resistant under hypoxic conditions. To investigate these questions GM38 and 3437T cells were suspended in growth media under either hypoxic (<10 p.p.m. oxygen) or aerobic (21% oxygen) conditions during exposure to $0.5 \mu g ml^{-1}$ of either MMC or PM for different periods of time. Again, a six-fold increase in resistance to MMC of 3437T compared to GM38 fibroblasts was observed under aerobic suspension conditions (Figure 3). As previously noted for other cell lines, MMC demonstrated increased toxicity towards both GM38 and 3437T strains when exposures were conducted under hypoxic conditions. However, the MMC resistance demonstrated by 3437T relative to normal fibroblasts under aerobic conditions was lost under hypoxic conditions. An increase in sensitivity to MMC under hypoxic conditions was observed for 3437T compared to GM38 cells, but the difference was small (1.4-fold).

As shown in Figure 4, exposure of these cell strains in suspension to PM under either aerobic or hypoxic conditions yielded results qualitatively similar to those for MMC. As observed in the monolayer system (Figure 2), PM was less toxic than MMC towards both cell lines under aerobic conditions. Also, 3437T was more resistant than GM38. PM was more toxic to both cell strains under hypoxic conditions, and the resistance of 3437T, relative to GM38, disappeared under such conditions. The toxicity of PM under hypoxic conditions was almost identical to that observed for MMC at equimolar levels, in contrast to the increased toxicity observed in rodent cell lines under hypoxic conditions (Keyes *et al.*, 1985*a*,*b*; Fracasso & Sartorelli, 1986).

**Figure 3** Survival of human fibroblasts in suspension as a function of exposure time to $0.5 \mu g ml^{-1}$ MMC under aerobic (open symbols) and hypoxic conditions (filled symbols). While strain 3437T is more resistant than strain GM38 to aerobic exposures, this selective resistance is lost under hypoxic conditions.

**Figure 4** Survival of human fibroblasts in suspension as a function of exposure time to $0.5 \mu g ml^{-1}$ PM under aerobic (open symbols) and hypoxic conditions (filled symbols). Strain 3437T is more resistant under aerobic conditions only.

Resistance to bioreductive alkylating agents may arise from enhanced repair of the resulting lethal lesions. Interstrand DNA cross-links are the apparent lethal lesion produced by both PM and MMC. To determine whether increased ability to repair DNA cross-links leads to the increased aerobic resistance of 3437T to these agents, response to cisplatin, a cross-linking agent which does not require metabolic activation, was investigated. The two cell strains were exposed to various concentrations of cisplatin in monolayers in the same fashion as described for MMC in Figure 1. Figure 5 demonstrates that GM38 and 3437T were almost identical in their sensitivities to cisplatin, suggesting that enhanced repair of interstrand cross-links was not responsible for the resistant phenotype of 3437T.

The failure of 3437T to demonstrate enhanced resistance under hypoxic conditions suggested that its resistance might arise from an inability to activate these agents under aerobic conditions. Dicumarol is an agent which inhibits the aerobic

**Figure 5** Relative plating efficiency of GM38 (filled symbols) and 3437T (open symbols) human skin fibroblasts after a 1 hour aerobic exposure in monolayer to various concentrations of cisplatin. Points are averages of at least three experiments ±s.e.m.

**Figure 6** Relative plating efficiency of GM38 and 3437T cells after 1 hour exposures to various MMC concentrations in the presence or absence of 1.0 or 2.0 mM dicumarol. Addition of dicumarol had no effect at either level on the survival of 3437T, but protected GM38. Exposures were conducted under aerobic conditions in monolayer.

activation of MMC and PM, probably through its inhibition of DT-diaphorase, an enzyme involved in the aerobic activation of these compounds (Keyes *et al.*, 1984). Dicumarol does not alter drug uptake under aerobic conditions in mouse EMT6 cells (Keyes *et al.*, 1986). If deficient aerobic activation was a cause of resistance it would be expected that blocking a potential activation pathway with dicumarol could increase resistance of the GM38 fibroblasts while having little or no effect on the sensitivity of 3437T fibroblasts. GM38 and 3437T cells were exposed in monolayers to various concentrations of MMC under aerobic conditions in the absence or presence of 1.0 or 2.0 mM dicumarol. Dicumarol increased the resistance of the control fibroblasts to MMC but had no effect on the resistance of the 3437T cells (Figure 6). Below MMC concentrations of 1 $\mu g ml^{-1}$ the protective effect for GM38 was identical for either 1 or 2 mM dicumarol. At higher MMC concentrations the protective effect was greater for 2 mM dicumarol.

### Discussion

Preferential hypoxic cell toxicity of bioreductive alkylating agents has been demonstrated previously in rodent cells (Kennedy *et al.*, 1980; Marshall & Rauth, 1986). While it has been assumed that such selectivity could also be obtained in human cells, no increase in cytotoxicity of MMC under hypoxic conditions was observed in a series of human tumour samples (Ludwig, 1984). In the present work, non-transformed, human skin fibroblasts were significantly more sensitive to both MMC and PM under hypoxic than under aerobic conditions (Figures 3 and 4). It would appear, therefore, that while preferential hypoxic cytotoxicity occurs in cells of human origin, the ability to demonstrate this characteristic may depend on several factors. Variations between cell lines and differences in actual oxygen concentration under hypoxic conditons may alter the degree of preferential toxicity observed (Marshall & Rauth, 1986; Gupta & Constanzi, 1987). Other variables which may also be important in such comparisons include drug concentration, duration of drug exposure, cell density, presence or absence of agents which selectively modify cytotoxicity and metabolic state of the cells (Rockwell, 1986; Marshall & Rauth, 1986; Gupta & Constanzi, 1987).

Development of drug resistance in a tumour may be a significant barrier to curability. It is notable, therefore, that the aerobic resistance to either MMC or PM demonstrated by the cell strain 3437T, compared to the normal cell strain GM38, is absent under hypoxic conditions (Figures 3 and 4). Clinically, bioreductive alkylating agents might be combined with radiation in order to increase local control of specific solid tumours in which radioresistant hypoxic populations are a limiting factor (Rockwell & Sartorelli, 1989). The loss of MMC resistance under hypoxic conditions would suggest that drug resistance of aerobic cell populations may not always be a limitation to this form of combined therapy. It also suggests that the activity of bioreductive alkylating agents should be examined under hypoxic, as well as aerobic, conditions. MMC-resistant variants of a human colonic carcinoma have recently been derived and characterised under aerobic conditions (Long *et al.*, 1984; Dorr *et al.*, 1987). Analogues of MMC have also been synthesised and characterised with respect to their cytotoxicity toward such resistant populations under aerobic conditions (Willson *et al.*, 1985; Chakrabarty *et al.*, 1986). It was therefore of interest to examine the activity of these analogues against a resistant cell strain under hypoxic conditions to determine whether preferential cytotoxicity could be obtained, especially if they are to be combined with ionising radiation for treatment of solid tumours.

Porfiromycin is one MMC analogue which has been found to be less toxic towards aerobic and at least as toxic towards hypoxic murine cells when compared to MMC (Keyes *et al.*, 1985; Fracasso & Sartorelli, 1986). In the present study it was found that PM was less toxic towards aerobic human cells than MMC, but equitoxic under hypoxic conditions. Cells which were resistant to MMC under aerobic conditions were similarly resistant to PM. Therefore, this analogue might demonstrate an enhanced therapeutic ratio, as an adjunct to radiation therapy, as a result of decreased toxicity towards normal, well-oxygenated tissue without loss in hypoxic cytotoxicity.

Cellular resistance to MMC and PM may occur by at least four different mechanisms. (1) Mutants have been isolated with are deficient in the repair of DNA–DNA cross-links and exquisitely sensitive to MMC (Thomson *et al.*, 1980; Meyn *et al.*, 1982), suggesting that enhanced repair of DNA cross-links could lead to increased drug resistance. (2) Bioreductive alkylating agents require metabolic activation before demonstrating cytotoxicity, so that a deficiency in the enzymatic pathways responsible for such activity would also lead to less cellular damage and apparent drug resistance. (3) Altered levels of intracellular protective agents such as glutathione. (4) Decreased drug transport arising from membrane alterations may protect against cytotoxicity.

If MMC resistance of the 3437T cells was to result from enhanced repair of DNA cross-links, resistance might also be observed upon exposure to other cross-linking agents. Resistant and control human fibroblasts utilised in these experiments demonstrated no difference in their sensitivity to cisplatin, another chemotherapeutic agent known to introduce interstrand DNA cross-links. While both MMC and cisplatin produce a variety of lesions, such as DNA and protein adducts, DNA–protein cross-links and both inter- and intrastrand DNA cross-links, interstrand DNA cross-links are closely associated with observed cytotoxicity (Pinto & Lippard, 1985; Plooy *et al.*, 1985). The observation that mutants sensitive to both MMC and cisplatin are also deficient in the repair of interstrand DNA cross-links suggests also that the modes of toxicity of these agents may be similar (Meyn *et al.*, 1982). In other studies, there were no differences in cytotoxicity between 3437T and GM38 after exposure to another cross-linking agent, MNU (Paterson *et al.*, 1986). While each of these agents introduces DNA–DNA cross-links, only MMC and PM require reductive activation before generation of reactive, DNA-damaging species. It has been demonstrated previously that the MMC resistant 3437T cells accumulate fewer DNA cross-links than a comparable normal cell strain after an equivalent dose of MMC (Paterson *et al.*, 1986). Normal sensitivity to other cross-linking agents suggests that this is not due to an increase in the repair of cross-links. Together, these data suggest that increased repair of interstrand DNA cross-links is not the reason for the observed resistance to MMC and PM and suggest that deficient activation of these compounds may be responsible.

Cellular enzymes implicated in the activation of mitomycin compounds include xanthine oxidase, cytochrome c reductase and DT-diaphorase (NAD(P)H dehydrogenase) (Pan *et al.*, 1984; Keyes *et al.*, 1984). Exposure to either PM or MMC under hypoxic conditions increases the level of cytotoxicity in both cell strains to the same level, eliminating the resistant phenotype demonstrated by 3437T under aerobic conditions. These results suggest that MMC resistance in the 3437T cells results from deficient drug activation under aerobic, but not hypoxic, conditions. While the relative importance of each system under hypoxic and/or aerobic conditions remains unclear, DT-diaphorase appears to be involved in aerobic activation (Keyes *et al.*, 1984).

It was found that dicumarol, an inhibitor of DT-diaphorase, was able to modify the aerobic MMC sensitivity of normal, but not resistant, human fibroblasts. The relatively high concentrations of dicumarol used in these studies were based upon previous aerobic cell survival studies by Keyes *et al.* (1986) in which surviving fraction increased as dicumarol concentration increased from 0.03 to 1.0 mM. The observation that 2 mM dicumarol provided enhanced protective effects relative to 1 mM dicumarol only at higher MMC concentrations (Figure 6) may indicate a saturation of dicumarol's protective effect at low MMC concentrations. The poor solubility of dicumarol prevented investigation of this effect at higher concentrations. However, the ability of dicumarol to increase the level of aerobic resistance of the control but not the resistant cells suggested that a deficiency in DT-diaphorase (NAD(P)H dehydrogenase), an enzyme capable of 2-electron, aerobic reduction of quinone compounds (Iyanagi & Yamazaki, 1970), was partially responsible for the aerobic MMC resistance demonstrated by the 3437T cells. Indeed, initial measurements using the method of Benson *et al.* (1980) indicate that substantial levels of DT-diaphorase activity are present in GM38 cells ($1830 \pm 220$ nmol $min^{-1}$ mg $protein^{-1}$), with little activity being detected in the 3437T cells ($30 \pm 20$ nmol $min^{-1}$ mg $protein^{-1}$).

While dicumarol is a specific inhibitor of DT diaphorase (Ernster *et al.*, 1962), the concentration utilised in these experiments may also have some effect upon respiration (Conover & Ernster, 1962) and intracellular calcium homeostasis (Thor *et al.*, 1982). However, such alternations might be expected to increase rather than decrease the cytotoxic effects of MMC, as was found by Thor *et al.* (1982) in the exposure of rat hepatocytes to both dicumarol and menadione. These effects of dicumarol may be responsible for the increased hypoxic cytotoxicity of MMC observed by Fracasso & Sartorelli (1986), however, the present data are unable to address this question. An examination of the effects of dicumarol on the hypoxic cytotoxicity of MMC to strains GM38 and 3437T might provide further evidence to support this mechanism for increased toxicity.

Addition of dicumarol to GM38 during MMC exposures did not completely mimic the drug-resistant phenotype. This may suggest either that external addition of this agent does not completely inhibit intracellular DT-diaphorase activity, or that additional factors are involved in the aerobic resistance of 3437T. No over-expression of P-glycoprotein, a membrane protein associated with pleiotropic drug resistance (Kartner *et al.*, 1983), was observed in the 3437T cells (data not shown). It is possible, however, that another factor involved with aerobic drug transport may be altered in these cells (Taylor *et al.*, 1985; Willson *et al.*, 1984). Transport of these agents under hypoxic conditions appears to be more rapid than under aerobic conditions (Keyes *et al.*, 1987). Whether this is a result of enhanced diffusion gradients due to increased intracellular drug metabolism or differences in transport related to hypoxia is unclear. The current results would be consistent with a model in which MMC transport is different under aerobic versus hypoxic conditions, such that an alteration in an aerobic but not hypoxic transport mechanism(s) could occur.

Conflicting reports in the literature make it difficult to attribute the aerobic drug resistance to altered levels of intracellular protective agents (Shrieve & Harris, 1986; Geard & Georgsson, 1986). If gluthathione does modulate MMC and PM cytotoxicity it may be possible that increased levels play a role in the aerobic resistance observed in these cells.

While these observations provide evidence for the existence of at least two separate and distinct pathways of bioreductive activation under aerobic and hypoxic conditions, other factors which may also contribute to MMC resistance, such as altered transport or increased levels of intracellular protective agents, have not been eliminated. Detailed studies of DT-diaphorase levels and the possible contribution of altered drug transport and glutathione levels to MMC resistance in these cell strains are in progress.

This research was supported by the National Cancer Institute, Medical Research Council, Ontario Cancer Treatment and Research Foundation, Alberta Heritage Foundation for Medical Research and the US National Cancer Institute through contract NO1-CP-21029 (Basic) with the Clinical and Environmental Epidemiology Branches, NCI, Bethesda, Maryland.

### References

BENSON, A.M., HUNKELER, M.J. & TALALAY, P. (1980). Increase of NAD(P)H:quinone reductase by dietary antioxidants: possible role in protection against carcinogenesis and toxicity. *Proc. Natl Acad. Sci. USA*, **77**, 5216.

CHAKRABARTY, S., DANELS, Y.J., LONG, B.H., WILLSON, J.K.V. & BRATTAIN, M.G. (1986). Circumvention of deficient activation in mitomycin C-resistant human colonic carcinoma cells by the mitomycin C analogue BMY 25282. *Cancer Res.*, **46**, 3456.

CONOVER, T.E. & ERNSTER, L. (1962). DT Diaphorase II relation to respiratory chain of intact mitochondria. *Biochim. Biophys. Acta*, **58**, 189.

DORR, R.T., LIDDIL, J.D., TRENT, J.M. & DALTON, W.S. (1987). Mitomycin C resistant L1210 leukemia cells: association with pleiotropic drug resistance. *Biochem. Pharmacol.*, **36**, 3115.

ERNSTER, L., DANIELSON, L. & LJUNGGREN, M. (1962). DT Diaphorase I purification from the soluble fraction of rat-liver cytoplasm, and properties. *Biochim. Biophyss. Acta*, **58**, 171.

FRACASSO, P.M.. & SARTORELLI, A.C. (1986). Cytotoxicity and DNA lesions produced by mitomycin C and porfiromycin in hypoxic and aerobic EMT6 and Chinese hamster ovary cells. *Cancer Res.*, **46**, 3939.

GEARD, C.R. & GEORGSSON, M.A. (1986). Glutathione levels and cytotoxicity of a thiol activated alkylating agent in human and mouse cells. *Int. J. Radiat. Oncol. Biol. Phys.*, **12**, 1179.

GUPTA, V. & CONSTANZI, J.J. (1987). Role of hypoxia in anticancer drug-induced cytotoxicity for Ehrlich Ascites cells. *Cancer Res.*, **47**, 2407.

IYANAGI, T. & YAMAZAKI, I. (1970). One-electron-transfer reactions in biochemical systems. V: difference in the mechanism of quinone reduction by the NADH dehydrogenase and the NAD(P)H dehydrogenase (DT-diaphorase). *Biochim. Biophys. Acta.*, **216**, 282.

IYER, V.N. & SZYBALSKI, W. (1963). A molecular mechanism of mitomycin action: linking of complementary DNA strands. *Proc. Natl Acad. Sci. USA*, **50**, 355.

KARTNER, N., RIORDAN, J.R. & LING, V. (1983). Cell surface P-glycoprotein associated with multidrug resistance in mammalian cell lines. *Science*, **221**, 1285.

KENNEDY, K.A., ROCKWELL, S. & SARTORELLI, A.C. (1980). Preferential activation of mitomycin C to cytotoxic metabolites by hypoxic cells. *Cancer Res.*, **40**, 2356.

KENNEDY, K.A., SLIGAR, S.G., POLOMSKI, L. & SARTORELLI, A.C. (1982). Metabolic activation of mitomycin C by liver microsomes and nuclei. *Biochem. Pharmacol.*, **31**, 2011.

KEYES, S.R., FRACASSO, P.M., HEIMBROOK, D.C., ROCKWELL, S., SLIGAR, S.G. & SARTORELLI, A.C. (1984). Role of NADPH: cytochrome c reductase and DT-diaphorase in the biotransformation of mitomycin C. *Cancer Res.*, **44**, 5628.

KEYES, S.R., ROCKWELL, S. & SARTORELLI, A.C. (1985*a*). Enhancement of mitomycin C cytotoxicity to hypoxic tumor cells by dicoumarol *in vivo* and *in vitro*. *Cancer Res.*, **45**, 213.

KEYES, S.R., ROCKWELL, S. & SARTORELLI, A.C. (1985*b*). Porfiromycin as a bioreductive alkylating agent with selective toxicity to hypoxic EMT6 tumor cells *in vivo* and *in vitro*. *Cancer Res.*, **45**, 3642.

KEYES, S.R., ROCKWELL, S. & SARTORELLI, A.C.. (1986). Mechanistic studies on dicumarol-enhanced toxicity of mitomycin C and porfiromycin to hypoxic EMT6 tumour cells. *Proc. Am. Assoc. Cancer Res.*, **27**, 233.

KEYES, S.R., ROCKWELL, S. & SARTORELLI, A.C. (1987). Correlation between drug uptake and selective toxicity of porfiromycin to hypoxic EMT6 cells. *Cancer Res.*, **47**, 5654.

LONG, B.H., WILLSON, J.K.V., BRATTAIN, D.E., MUSIAL, S. & BRATTAIN, M.G. (1984). Effects of mitomycin C on human colon carcinoma cells. *J. Natl Cancer Inst.*, **73**, 787.

LUDWIG, C. (1984). Drug resistance of hypoxic tumour cells *in vitro*. *Cancer Treat. Rev.*, **11**, 173.

MARSHALL, R.S., KOCH, C.J. & RAUTH, A.M. (1986). Meaasurement of low levels of oxygen and their effect on respiration in cell suspensions maintained in an open system. *Radiat. Res.*, **108**, 91.

MARSHALL, R.S., PATERSON, M.C. & RAUTH, A.M. (1989). Aerobic resistance of a human cell strain to mitomycin C and porfiromycin is lost under hypoxic exposure conditions. *Biochem. Pharmacol.*, in the press.

MARSHALL, R.S. & RAUTH, A.M. (1986). Modification of the cytotoxic activity of mitomycin C by oxygen and ascorbic acid in Chinese hamster ovary cells and a repair-deficient mutant. *Cancer Res.*, **46**, 2709.

MEYN, R.E., JENKINS, S.F. & THOMPSON, L.H. (1982). Defective removal of DNA cross-links in a repair-deficient mutant of Chinese hamster ovary cells. *Cancer Res.*, **42**, 3106.

PAN, S.S., ANDREWS, P.A. & GLOVER, C.J. (1984). Reductive activation of mitomycin C and mitomycin C metabolites catalyzed by NADPH-cytochrome P450 reductase and xanthine oxidase. *J. Biol. Chem.*, **259**, 959.

PATERSON, M.C., MIDDLESTADT, M.V., WEINFELD, M., MIRZAYANS, R. & GENTNER, N.E. (1986). Human cancer-prone disorders, abnormal carcinogen response, and defective DNA metabolism. In *Radiation Carcinogenesis and DNA Alterations*, Burns, F.J., Upton, A.C. & Silini, G. (eds) p. 471. Plenum Press: New York.

PINTO, A.L. & LIPPARD, S.J. (1985). Binding of the antitumor drug *cis*-diamminedichloroplatinum(II) (cisplatin) to DNA. *Biochim. Biophys. Acta*, **780**, 167.

PLOOY, A.C.M., VAN DIJK, M., BERENDS, F. & LOHMAN, P.H.M. (1985). Formation and repair of DNA interstrand crosslinks in relation to cytotoxicity and unscheduled DNA synthesis induced in control and mutant human cells treated with *cis*-diamminedichloroplatinum(II). *Cancer Res.*, **45**, 4178.

RAHMOUNI, A. & LENG, M. (1987). Reaction of nucleic acids with *cis*-diamminedichloroplatinum(II): interstrand cross-links. *Biochemistry*, **26**, 7229.

ROCKWELL, S. (1983). Effects of mitomycin C alone and in combination with x-rays on EMT6 mouse mammary tumours *in vivo*. *J. Natl Cancer Inst.*, **71**, 765.

ROCKWELL, S. (1986). Effect of some proliferative and environmental factors on the toxicity of mitomycin C to tumour cells *in vitro*. *Int. J. Cancer*, **38**, 229.

ROCKWELL, S. & SARTORELLI, A.C. (1989). Mitomycin C and radiation. In *Interactions Between Antitumor Drugs and Radiation*, Hill, B. & Bellamy, A. (eds). CRC Press: Boca Raton, FL.

SHRIEVE, D.C. & HARRIS, J.W. (1986). Effects of glutathione depletion by buthionine sulfoximine on the sensitivity of EMT6/SF cells to chemotherapy agents or X radiation. *Int. J. Radiat. Oncol. Biol. Phys.*, **12**, 1171.

TAYLOR, C.W., BRATTAIN, M.G. & YEOMAN, L.C. (1985). Occurrence of cytosolic protein and phosphoprotein changes in human colon tumor cells with the development of resistance to mitomycin C. *Cancer Res.*, **45**, 4422.

THOMPSON, L.H., RUBIN, J.S., CLEAVER, J.E., WHITMORE, G.F. & BROOKMAN, K. (1980). A screening method for isolating DNA repair-deficient mutants of CHO cells. *Somatic Cell Genet.*, **6**, 391.

THOR, H., SMITH, M.T., HARTZELL, P., BELLOMO, G., JEWELL. S.A. & ORRENIUS, S. (1982). The metabolism of menadione (2-methyl-1,4-naphthoquinone) by isolated hepatocytes. *J. Biol. Chem.*, **257**, 12419.

WILLSON, J.K., LONG, B.H., CHAKRABARTY, S., BRATTAIN, D.E. & BRATTAIN, M.G. (1985). Effects of BMY 25282, a mitomycin C analogue, in mitomycin C-resistant human colon cancer cells. *Cancer Res.*, **45**, 5281.

WILLSON, J.K.V., LONG, B.H., MARKS, M.E., BRATTAIN, D.E., WILEY, J.E. & BRATTAIN, M.G. (1984). Mitomycin C resistance in a human colon carcinoma cell line associated with cell surface protein alterations. *Cancer Res.*, **44**, 5880.